# The use of indocyanine green for colorectal anastomoses: a systematic review and meta-analysis

**DOI:** 10.1308/rcsann.2024.0002

**Published:** 2024-09-24

**Authors:** L Borg, M Portelli, L Testa, P Andrejevic

**Affiliations:** ^1^ Mater Dei Hospital, Malta; ^2^ University of Malta, Malta

**Keywords:** colorectal, anastomosis, indocyanine green, surgery

## Abstract

**Introduction:**

Anastomotic leak is a relatively common and debilitating complication. Colorectal leak rates vary widely in the literature, ranging from 1% to 20%. In modern surgical practice, there is much emphasis on the use of indocyanine green (ICG). This is a fluorescent dye administered intravenously to locate and predict an adequate line of anastomosis. We sought to analyse the current literature and supporting evidence behind the use of ICG in the context of elective colorectal surgery.

**Methods:**

A literature search was conducted for papers published between January 1991 and December 2022 concerning the use of ICG in colorectal surgery. Data on anastomotic leak, overall complication rate, operative time and involvement of artificial intelligence (AI) were compared.

**Results:**

A total of 24 studies were selected, including 3 randomised controlled trials. There was an anastomotic leak rate of 4.3% in cases with ICG administration compared with 9.5% in the control group (*p*<0.00001). Seven studies mentioned overall complication rates. These were lower in the ICG cohort than in the control group (15.5% vs 24.5%). There was no significant correlation between ICG use and operative time (*p*=0.78). Five studies looked at AI, with results suggesting that use of AI leads to much better accuracy in ICG metric analysis. However, the current literature is still inconclusive.

**Conclusions:**

While there is strong evidence behind ICG use in the existing literature, more randomised controlled trials are required for better recommendations. AI in ICG metric interpretation has proved to be difficult owing to interpatient variability. Nevertheless, new data suggest better understanding and standardisation.

## Introduction

Anastomotic leak remains a severe complication in colorectal surgery and the incidence rate varies widely in the literature, ranging from 1% to 20%.^1^ Indocyanine green (ICG) is a fluorescent dye that is injected intravenously, with the resulting vascular patterns and dye uptake enhancing understanding of anatomy and the associated vasculature.^2^ When coupled with near infrared light, this technique has been given multiple applications in various disciplines, most notably in hepatobiliary and colorectal surgery.^3^ In this study, we look at its use in colorectal surgical practice, with a focus on the predictive value of anastomotic line placement. Anastomotic leaks are relatively common, complex to manage and a significantly morbid complication of any respective procedure.^4^ Minimising the risk of anastomotic leak would improve the quality of care on an individual level as well as the burden of care requirements with respect to hospital-associated resources necessary for care on a macroeconomic level.

The main aim of the study was to review the published literature concerning the use of ICG, with emphasis on the predictive value for anastomotic leak following colorectal surgery. A secondary aim was to also review what role artificial intelligence (AI) plays in data analysis and interpretation.

## Methods

A literature search was carried out on the PubMed^®^, MEDLINE^®^, Cochrane Library and Google Scholar™ databases using the Medical Subject Headings “indocyanine green”, “artificial intelligence”, “colorectal” and “anastomosis”, combined with the Boolean operators “OR” and “AND” as well as wildcards such as “+” and “?”. Studies published between 1 January 1991 and 31 December 2022 on the relevant topic were identified and analysed. Inclusion criteria for our clinical review included established guidelines, randomised controlled trials (RCTs) and non-RCTs with a follow-up duration of over two months. The literature search was performed independently by all three authors. The compiled list of results was then analysed and all duplicates were removed from the list. From the remaining studies, qualitative studies and individual case reports were excluded. All RCTs and any studies that evaluated AI usage quantitatively were included.

All data were compiled using RevMan 5.3 (Nordic Cochrane Centre, Copenhagen, Denmark) as well as SPSS^®^ Statistics (IBM, New York, US) and the review included cross-study comparison of the reported data. A database was created of individual odds ratios and mean differences with 95% confidence intervals (CIs). This systematic review was performed in accordance with the PRISMA (Preferred Reporting Items for Systematic reviews and Meta-Analysis) guidelines.^5^ Risk of bias for the individual studies was evaluated using the Cochrane RoB 2 tool for randomised controlled trials (RCTs)^6^ and the ROBINS-I tool for non-randomised studies.^7^

## Results

### Study characteristics

**Figure 1:**
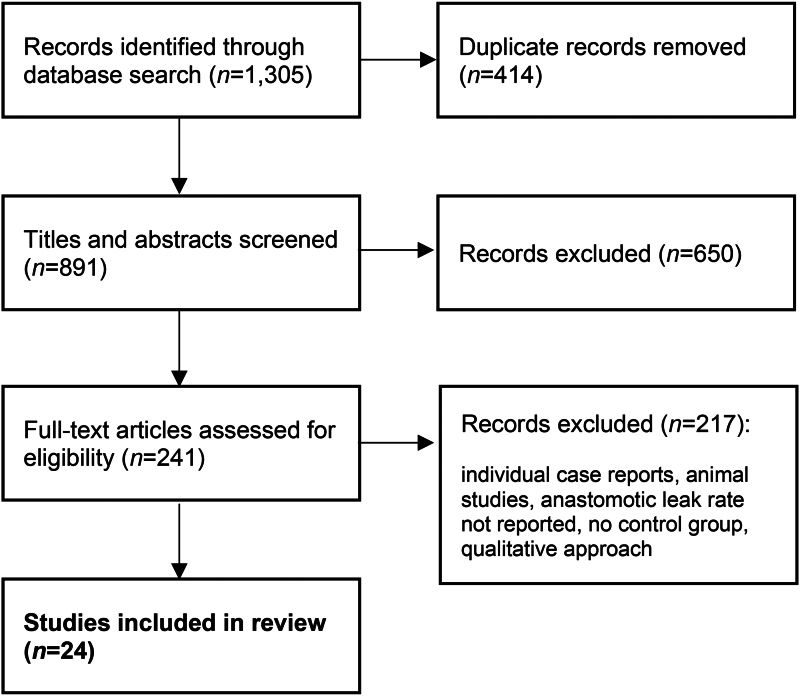
Flowchart of study selection

A total of 1,305 records were identified from the initial literature search, with 24 of these selected for this review (Figure 1). The final dataset comprised 24 studies, including 3 RCTs.^8–31^ An overview of these is given in Table 1. The papers provided data from 12 different countries, including 11 studies from Europe (Italy, Spain, Czech Republic, Russia, UK, Germany, Switzerland), 4 from the US and 9 from Asia (Japan, China, South Korea, Hong Kong). The papers were published between 2010 and 2021, with sample sizes ranging from 38 to 657. The total number of patients was 5,877 (2,565 in the ICG group and 3,312 in the control group).

**Table 1:** Summary of included studies

Study	Country	Data range	Study design	Total sample size	ICG group	Controlgroup
Alekseev, 2020^8^	Russia	2018–2019	RCT	377	187	190
Bonadio, 2020^9^	Italy	ICG: 2015–2017Control: 2014–2015	Retrospective study	66	33	33
Boni, 2017^10^	Italy	ICG: 2014–2015Control: 2012–2013	Retrospective study	80	42	38
Brescia, 2018^11^	Italy	2014–2017	Retrospective study	182	75	107
De Nardi, 2020^12^	Italy	2016–2017	RCT	240	118	122
Dinallo, 2019^13^	UK	2013–2016	Retrospective study	554	234	320
Hasegawa, 2020^14^	Japan	ICG: 2016–2017Control: 2007–2016	Retrospective study	420	141	279
Impellizzeri, 2020^15^	Italy	2014–2019	Retrospective study	196	98	98
Ishii, 2020^16^	Japan	ICG: 2017–2018Control: 2014–2018	Retrospective study	220	116	104
Jafari, 2021^17^	US	2015–2017	RCT	347	178	169
Jafari, 2013^18^	US	2011–2012	Retrospective study	38	16	22
Kim, 2017^19^	Korea	ICG: 2013–2016Control: 2010–2016	Retrospective study	657	310	347
Kin, 2015^20^	US	2005–2012	Retrospective study	346	173	173
Kojima, 2020^21^	Japan	Not mentioned	Retrospective study	54	27	27
Kudszus, 2010^22^	Germany	1998–2008	Retrospective study	402	201	201
Mizrahi, 2018^23^	US	ICG: 2015–2016Control: 2013–2015	Retrospective study	60	30	30
Otero-Piñeiro, 2021^24^	Spain	ICG: 2016–2018Control: 2011–2016	Retrospective study	284	80	204
Ren, 2018^25^	China	2015–2017	Retrospective study	55	32	23
Ris, 2018^26^	Switzerland	Not mentioned	Retrospective study	455	90	365
Skrovina, 2020^27^	Czech Republic	2015–2017	Retrospective study	100	50	50
Tsang, 2020^28^	Hong Kong	2018–2019	Retrospective study	131	63	68
Wada, 2017^29^	Japan	ICG: 2013–2016Control: 2009–2016	Retrospective study	149	48	101
Watanabe, 2020^30^	Japan	2014–2017	Retrospective study	422	211	211
Zhou, 2019^31^	China	2017–2019	Retrospective study	42	12	30
ICG = indocyanine green; RCT = randomised controlled trial						

**Figure 2:**
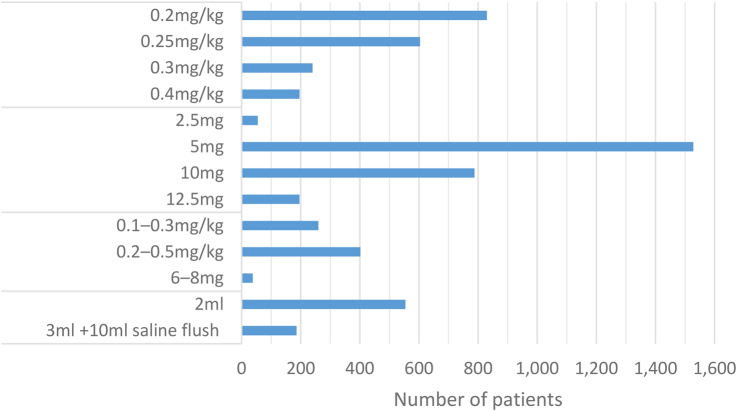
Frequency of use of various dosages of indocyanine green^8–31^

**Figure 3:**
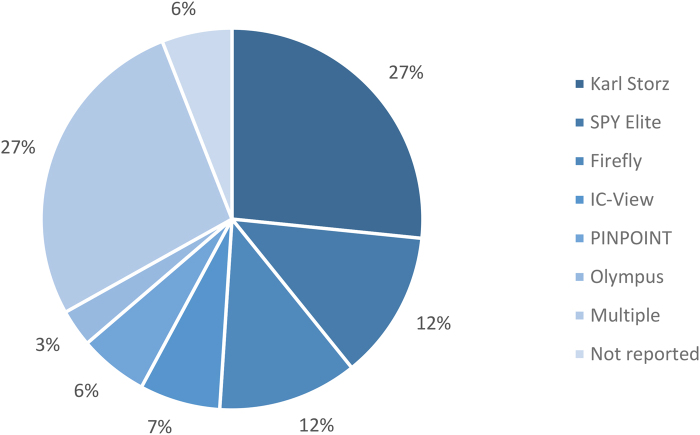
Imaging systems used in the included studies^8–31^

The most common surgical procedure performed was rectal surgery, with the majority of cases involving laparoscopic anterior resection of the rectum. Studies used different dosages of ICG as well as different imaging systems (Figures 2 and 3).

Risk of bias was assessed with the RoB 2 and ROBINS-I tools.^6,7^ Two of the studies included in this review were deemed to have a moderate risk of bias.^17,25^ The remaining 22 studies were graded as having a low risk of bias.

### Anastomotic leak and overall complications

**Figure 4:**
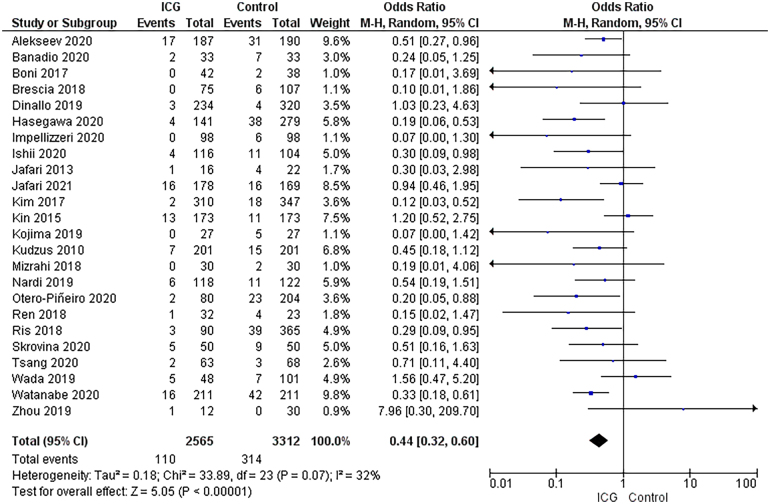
Forest plot illustrating the correlation between indocyanine green use and anastomotic leak^8–31^

There were 110 cases of anastomotic leak reported among the 2,565 patients in the ICG group (4.3%).^8–31^ In contrast, 314 of the 3,312 patients in the control group had anastomotic leak (9.5%). This difference was statistically significant (odds ratio: 0.44, 95% CI: 0.32–0.60, *p*<0.00001) (Figure 4).

Seven studies also reported rates of overall complications.^8,19,24,27,29–31^ In the ICG cohort, there were 139 cases out of 898 (15.5%) with documented evidence of complications. The control group had 278 such cases out of 1,133 (24.5%).

### Operative time

**Figure 5:**
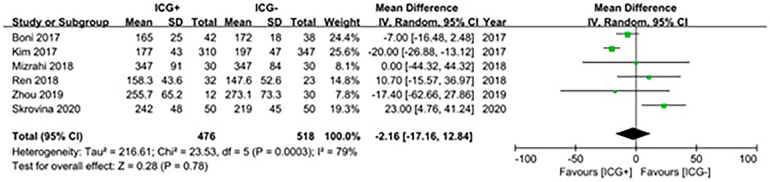
Forest plot illustrating the correlation between indocyanine green use and operative time^10,19,23,25,27,31^

Six studies were included in a meta-analysis comparing operative time for the ICG and control groups.^10,19,23,25,27,31^ The meta-analysis for operative time did not show any statistical significance (mean difference: −2.16 minutes, 95% CI: −17.16 minutes to 12.84 minutes, *p*=0.78) (Figure 5).

### Change of surgical plan

**Figure 6:**
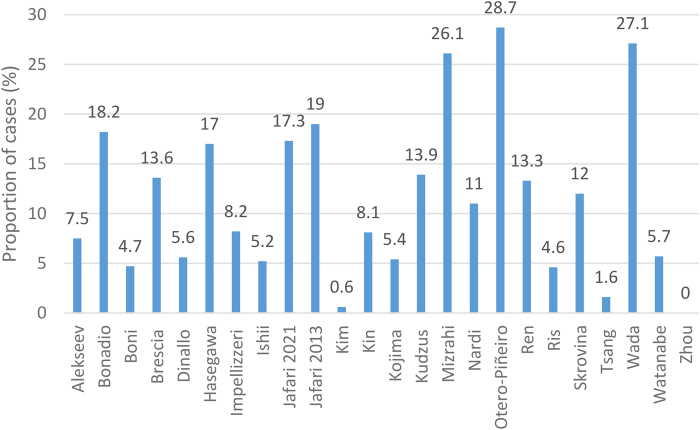
Proportion of cases in which use of indocyanine green changed surgical planning^8–31^

Across the dataset, all 24 studies looked at instances where use of ICG changed the positioning of an anastomotic placement.^8–31^ The proportion of cases where ICG assessment resulted in changes in anastomotic placement ranged from 0%^31^ to 28.7%^24^ (mean: 11.4%, standard deviation: 8.2pp, median: 9.6%, 95% CI: 11.2% to 11.6%) (Figure 6).

### Artificial intelligence

AI integration was explored in five of the studies included in this review.^8,12,24,28,29^ (Park *et al* further discussed real-time microcirculation analysis [50 cases in a training patient group and 15 cases in a test patient group] although their study did not compare ICG administration with a control group.)^32^ All five studies suggest that AI can play an important role in the interpretation of fluorescence intensity. The studies plotted different metric parameters on an ICG intensity slope against time. The metrics analysed included the maximum optic change in ICG fluorescence intensity (F_max_) and the time taken to reach F_max_ (T_max_) as well as half T_max_ (T_½ max_). The time ratio (TR) represents the analysis of the rising slope (RS) in the trend and is expressed as T_½ max_/T_max_. RS is consequently calculated as F_max_/T_max_.^32^ The results of the five studies indicate that TR is the most useful metric for ICG prediction.^8,12,24,28,29^

Kitaguchi *et al* quoted a similar result with an overall AI accuracy rate of 90.1%.^33^ This was a multicentre retrospective study looking at 300 cases (compared with the 65 patients in the study by Park *et al*).^32^ The paper by Kitaguchi *et al*, however, highlights phase recognition mean accuracy rather than comparing success rates specifically in perfusion (i.e. it is looking at AI in general in colorectal surgery and not completely in relation to ICG).^33^ They focused on sigmoid resections and high anterior resections to train and test AI models on phase and action recognition.

Like most AI-based phase sequencing, Kitaguchi *et al* divided videos into nine independent sequences.^33^ Actions were classified as dissection (actions for tissue separation), exposure (actions for tissue plane definition) and ‘other’ actions (e.g. cleaning the camera, colon transection and other extracorporeal actions). This was carried out under the supervision of two colorectal surgeons and created a dataset comprising over 82 million frames. The results showed an overall phase classification accuracy of 81.0%, with an accuracy of 83.2% for distinguishing dissection from exposure actions and an accuracy of 98.5% for recognising ‘other’ actions.

As the second largest study published with regard to AI use in colorectal surgery, the paper by Park *et al* looked directly at ICG use.^32^ Previous data suggest that one of the limitations of AI-mediated use of ICG is that of interpatient anatomical variation relating to microcirculation. Nevertheless, using T_½ max_, TR and RS, Park *et al* employed AI learning that was unsupervised by a surgeon. A cluster of over 10,000 ICG curves were analysed using AI, resulting in 25 reproducible patterns for which receiver operating characteristic curve analysis was undertaken and exact values for area under the curve (AUC) were calculated. The AUC for the AI model (0.842) indicated a higher accuracy than for T_½ max_ (0.750), TR (0.734) and RS (0.677). This was used to create a colour map of red, green and blue areas for classifying the grade of vascularisation.

Park *et al* compared AI with analogue ICG interpretation, concluding that AI was generally superior to conventional parameters, with rates of 87% for accuracy, 100% for recall, 60% for precision and 75% for F1 score (a measure of the harmonic mean of precision and recall).^32^ AI ranked second only in terms of precision (where TR achieved 67%).

## Discussion

Anastomotic leak is considered a relatively common and morbid complication for any individual undergoing colorectal surgery. It directly increases mortality rates and length of hospital stay.^13^ It also increases consumption of hospital resources and decreases postoperative patient mobility, contributing to a poor surgical outcome on multiple levels.^34^ A major pathophysiological predictor of success of anastomosis is that of perfusion quality. Anastomotic ischaemia plays a vital role as a predictive factor of anastomotic leak.^35^ Our literature review demonstrates a statistically strong association between perfusion-related ICG imaging and a reduction in anastomotic leakage. Use of ICG allows real-time assessment of intestinal perfusion, leading to a more objective definition of a planned transection line.^14^ This is underscored by the anastomotic leak rates of 4.3% and 9.5% for the ICG group and control group respectively.

Our study also noted a reduction in overall complication rates with use of ICG, from 24.5% to 15.5%. Further analysis of the association between ICG imaging and operative time showed no statistical significance, indicating that ICG use does not prolong operative time. This can potentially be explained by earlier confirmation of integrity of anastomosis. This suggests that in the future, with more efficient methods of analysis and interpretation, this practice can become routine throughout gastrointestinal surgery.^36^

Another issue is that of data analysis and interpretation. Multiple methods of analysis of fluorescence have been used to date, with multiple dosages and different schools of thought regarding analysis. Wada *et al* highlighted this by focusing on different fluorescence methods: infrared mapping variations and the respective predictive value.^29^ The most acceptable metrics to date are F_max_ and T_max_, representing the amount of change in ICG fluorescence intensity and the time taken to reach that point respectively.^18^ A cut-off slope value for F_max_ of 52AU seems to be a commonly cited threshold to reach acceptable agreement between experienced consultants.^20^

Interestingly, modern surgery is also attracting the incorporation of AI with AI-augmented risk map scoring as well as direct comparisons for different criteria. The study by Park *et al* is the only one to directly compare the effectiveness of AI with traditional metric standard analysis, using real-time video analysis, ultrasonic Doppler velocimetry for larger vessels and near infrared metrics to predictably compare the plotting of time and fluorescence intensity on the known ICG slope curve.^32^ This was used to classify areas as safe, intermediate or dangerous, enabling the data to be compared with retrospective anastomotic leak rates. The results from their study are reassuring and indicate the highest accuracy of assessment. Nevertheless, the AI group was limited to only 15 patients owing to the complex methodology.

### Study limitations

This study retains several limitations. Most of the data were retrospective, only three RCTs having been reported to date.^8,12,17^ Despite this, the data are clearly precise, as shown by the CI overlap and forest plot analysis. A second limitation is the inconsistency in study definitions. In certain studies, such as that by Boni *et al*, the definition of anastomotic leakage was restricted to confirmed evidence of disruption by water-soluble enema findings or computed tomography.^10^ In contrast, certain studies included abscess formation, enteric fistulation and similar anastomotic leak-associated complications without direct visualisation of the leak.^14,16,26^ A third (and perhaps significant) limitation of this review is the inconsistency found in ICG dosages and imaging mechanisms (Figures 2 and 3).

## Conclusions

ICG fluorescence angiography has a very strong predictive value in preventing anastomotic leak after colorectal surgery. Fluorescence is changing surgical practice for various techniques and across different subspecialties.^11^ However, more RCTs are required to weigh the evidence and provide direct recommendations for routine use. This also applies to other postoperative complications. The current literature indicates that there is minimal to no effect of ICG use on operative time. AI will play an important role in the future of ICG-mediated assessment algorithms, with limited preliminary published data suggesting that accuracy of AI is superior to traditional metric analysis. Further studies are required to investigate this further, with no specific methods yet agreed on by lower gastrointestinal surgeons/specialists.
